# Agreement of Potassium, Sodium, Glucose, and Hemoglobin Measured by Blood Gas Analyzer With Dry Chemistry Analyzer and Complete Blood Count Analyzer: A Two-Center Retrospective Analysis

**DOI:** 10.3389/fmed.2022.799642

**Published:** 2022-04-01

**Authors:** Hongxiang Xie, Shiyu Lv, Sufeng Chen, Zhenzhen Pang, Deli Ye, Jianzhuang Guo, Wanju Xu, Weidong Jin

**Affiliations:** ^1^Laboratory Medicine Center, Department of Clinical Laboratory, Zhejiang Provincial People's Hospital (Affiliated People's Hospital, Hangzhou Medical College), Hangzhou, China; ^2^Department of Clinical Laboratory Medicine, The First Affiliated Hospital of Shandong First Medical University and Shandong Provincial Qianfoshan Hospital, Shandong Medicine and Health Key Laboratory of Laboratory Medicine, Jinan, China

**Keywords:** blood gas analysis, electrolytes, glucose, hemoglobin, concordance analysis

## Abstract

**Background:**

Blood gas analyzers (BGAs) and dry biochemistry analyzers for potassium and sodium are based on direct electrode methods, and both involve glucose oxidase for glucose detection. However, data are lacking regarding whether the results of the two assay systems can be used interchangeably. In addition, there remains controversy over the consistency between BGA-measured hemoglobin and complete blood count analyzer data. Here, we compared the consistency of sodium, potassium, glucose, and hemoglobin levels measured by BGA and dry chemistry and complete blood count analyzers.

**Methods:**

Data from two teaching hospitals, the Zhejiang Provincial People's Hospital (ZRY) and the Qianfoshan Hospital (QY), were retrospectively analyzed based on dry biochemistry and complete blood count analyzer results as the reference system (X) and BGA as the experimental system (Y). Plasma was used for biochemical analysis at the ZRY Hospital, and serum at the QY Hospital. Paired data from the respective hospitals were evaluated for consistency, and biases between methods were assessed by simple correlation, Passing–Bablok regression, and Bland–Altman analyses.

**Results:**

The correlations of potassium, sodium, glucose, and hemoglobin measured by BGA and dry biochemistry and complete blood count analyzers were high, at 0.9573, 0.8898, 0.9849, and 0.9883 for the ZRY Hospital and 0.9198, 0.8591, 0.9764, and 0.8666, respectively, for the QY Hospital. The results of Passing to Bablok regression analysis showed that the predicted biases at each medical decision level were within clinically acceptable levels for potassium, sodium, glucose, and hemoglobin at the ZRY Hospital. Only the predicted bias of glucose was below the clinically acceptable medical decision levels at the QY Hospital, while potassium, sodium, and hemoglobin were not. Compared with the reference system, the mean bias for BGA measurements at the ZRY Hospital was −0.08 mmol/L (95% confidence interval [CI] −0.091 to −0.069) for potassium, 1.2 mmol/L (95% CI 1.06 to 1.42) for sodium, 0.20 mmol/L (95% CI 0.167 to 0.228) for glucose, and −2.8 g/L for hemoglobin (95% CI −3.14 to −2.49). The mean bias for potassium, sodium, glucose, and hemoglobin at the QY Hospital were −0.46 mmol/L (95% CI −0.475 to −0.452), 3.7 mmol/L (95% CI 3.57 to 3.85), −0.36 mmol/L (95% CI −0.433 to −0.291), and −8.7 g/L (95% CI −9.40 to −8.05), respectively.

**Conclusion:**

BGA can be used interchangeably with plasma electrolyte results from dry biochemistry analyzers but does not show sufficient consistency with serum electrolyte results from dry biochemistry analyzers to allow data interchangeability. Good consistency was observed between BGA and plasma or serum glucose results from dry biochemistry analyzers. However, BGA-measured hemoglobin and hematocrit assay results should be treated with caution.

## Introduction

Routine laboratory tests, such as potassium (K), sodium (Na), glucose (Glu), and hemoglobin (Hb) levels, are essential parameters in determining appropriate treatments for patients, especially those who are critically ill (1–4). Both hyperkalemia and hypokalemia are associated with poor clinical outcomes: In particular, hyperkalemia can induce or exacerbate arrhythmias and is associated with significantly increased mortality (1, 5). Therefore, severe hyperkalemia is a clinical emergency that requires immediate medical intervention. Similarly, sodium disorders are associated with increased morbidity and mortality (2). Hyponatremia can lead to permanent neurological damage and requires urgent treatment when patients present with severe or acute symptoms (e.g., altered mental status or seizures) (6). Blood glucose disorders, particularly severe hypoglycemia, are also associated with significant morbidity and mortality risk and are considered an independent risk factor for mortality in critically ill patients, so that strategies for early recognition are important to improve patient prognosis (3, 7). Hemorrhage is one of the most common causes of death in patients with severe trauma and the most common preventable cause of early trauma death (8). In addition to the patient vital signs, hemoglobin level is a key factor in determining the need for blood transfusion. Inaccurate hemoglobin findings may lead to serious adverse clinical consequences in certain emergencies (4).

In China, the laboratory departments of large hospitals generally have both dry and wet biochemistry analyzers as well as multiple complete blood count analyzers to provide testing for outpatient clinics and wards, respectively. Compared with traditional testing instruments, blood gas analyzers (BGAs) can directly test arterial or venous blood to obtain Na, K, Glu, and Hb measurements within minutes (9). Owing to their advantages of short testing time, easy operation, and low blood consumption, BGAs have been widely used in emergency testing departments. Both BGAs and dry chemistry are based on direct Ion-Selective Electrode (ISE), but there are important differences in the detail of chemical analysis methodology that may contribute to analytical variation (10). Taking Na as an example, dry chemistry analyzers require plasma/serum, and employ “dry-slide” technology in which plasma/serum Na activity generates an electrical potential across a static liquid junction situated on a paper bridge. By contrast, BGAs measurements are performed on whole blood with the ISE immersed on the plasma side of a red cell exclusion grid. Na activity generates a potential between the electrodes while fresh KCl solution is pumped continuously past a dynamic liquid junction (10). For Glu and Hb testing, BGAs uses testing principles different from those of dry biochemistry and complete blood count analyzers, which may also lead to differences in test results (11, 12).

How physicians or patients should interpret these discrepancies and the consistency and comparability of the results between different testing systems remain unknown. Although previous literature has compared BGA and central laboratory biochemistry analyzers, most of the latter tests for electrolytes employ indirect ion-selective electrode (indirect ISE) and relatively limited numbers of samples were compared, with varying results (9, 13, 14). Moreover, very limited data are available regarding the consistency between the results of BGA and dry biochemistry analyzers when used together in the emergency laboratory setting. The present study refers to the EP9-A3 document published by the Clinical and Laboratory Standards Institute (CLSI) (15) for a methodological comparison and bias assessment of several BGA assays (Na, K, Hb, Glu) to the same assays from other testing devices in the emergency laboratory setting. This two-center retrospective analysis aimed to provide clinicians and patients with correct data and reasonable analytical results to help them make proper clinical judgments.

## Data and Methods

### Sample Collection and Testing

The Zhejiang Provincial People's Hospital (ZRY Hospital) used an ABL800 FLEX BGA (Radiometer, Denmark) to test arterial whole blood. An arterial blood collection needle (Radiometer, Denmark) with dry electrolyte-balanced lithium-heparin anticoagulant was used to draw ~1.6 mL of patient arterial blood. These samples were then sent to the Laboratory Department, where they were tested within 15 min of receipt by the Emergency Laboratory Department. Venous blood was inverted eight times and then centrifuged at 3,000 r/min for 10 min to separate the plasma; a VITROS 5600 dry biochemical analyzer (Johnson & Johnson, USA) was used for electrolyte or Glu analysis, which was completed within 40 min. An BC-6900 fully automated hematology analyzer (Mindray Co., Ltd., China) was used for Hb measurement of EDTA-K2 anticoagulant venous blood. Arterial and venous blood samples were collected by trained medical personnel. The reagents provided by the manufacturer were used and all samples were tested in strict accordance with the appropriate instruments, reagents, and standard operation procedure (SOP) of the department. The instruments were subjected to daily in-house quality control and regular participation in an external quality assessment program to ensure the accuracy of the test results.

The First Affiliated Hospital of Shandong First Medical University (QY Hospital) used a VITROS350 dry biochemical analyzer (Johnson & Johnson Inc, USA), a GEM3500 BGA (Instrumentation Laboratory, USA), and an XN-9000 complete blood count analyzer (Sysmex, Japan). The hospital also used electrolyte-balanced dry heparin lithium anticoagulant arterial blood gas needles (Becton Dickinson, USA) to collect arterial blood for blood gas analysis, while venous blood serum was collected for biochemical analyses (instrument parameters were given in Supplementary Table S1).

### Data Collection

We retrospectively analyzed data in the Laboratory Information System (LIS) of the ZRY and QY Hospitals. The databases of both hospitals contained patient demographic, diagnostic, and laboratory analysis data. The laboratory analysis data included the date and time of sample collection and delivery to the testing department, as well as the time of sample testing. Sample data from blood gas analysis were paired with samples from biochemistry and/or hematology analyzers based on the following: samples collected at approximately the same time and that arrived at the testing department at approximately the same time. We excluded data from patients whose samples were collected more than 10 min apart; patients under 18 years of age; patients with hemolysis, jaundice, and lipemia; patients with repeated laboratory results. The study was approved by the ethics committees of the study sites.

### Method Comparison and Bias Assessment

Outlier tests were performed by using generalized extreme studentized deviate (ESD) according to the CLSI-EP9-A3 guidelines. We used the Passing–Bablok (PB) model to fit the regression equation, which assumes random errors in both measurement processes. The testing results of dry chemistry and whole blood cell analyzers were used as reference system (X) while the testing results of BGA were used as test system (Y). Based on the regression equation, the 95% confidence interval (CI) and bias at medical decision levels (MDL) (16) were calculated for each parameter. The acceptability criteria/acceptance limits (ALs) according to the US Clinical Laboratory Improvement Amendment Act (CLIA88) were used to determine the clinical significance of the bias (17). The following biases were considered acceptable according to the analytical quality requirements of the CLIA88 competency ratio: Na, ± 4 mmol/L; K, ± 0.5 mmol/L; Glu, ± 10%; and Hb, ± 7%. The mean bias and 95% limits of agreement (LoA) between the two measurements were further assessed using Bland–Altman plots. The results obtained from the dry chemistry biochemistry and complete blood count analyzers were used as the “gold standard” for comparisons.

### Data Statistics

The statistical analyses were performed using MedCalc version 19.0. The normality of the data distributions was determined by Kolmogorov–Smirnov tests, with normally distributed data expressed as mean and standard deviation, and Pearson correlation used for correlation analysis. Non-normally distributed data were expressed as medians and percentile, and Spearman correlation was used for correlation analysis. A coefficient >0.8 indicated a strong correlation and *P* < 0.05 indicated a statistically significant difference.

## Results

The K, Na, Glu, and Hb levels measured by different instruments in the emergency laboratory departments of the two teaching hospitals are shown in Table 1. The correlation coefficients and their 95% CIs indicate the degree of correlation between the paired samples. The K, Na, and Glu levels measured by BGA at the ZRY Hospital were highly correlated with those measured by the dry biochemistry analyzer, and the Hb level measured by BGA was highly correlated with that measured by the complete blood cell analyzer, with correlation coefficients of 0.9573, 0.8898, 0.9849, and 0.9883 for K, Na, Glu, and Hb, respectively. Similar results were observed in the QY Hospital, where the correlation coefficients between the BGA, dry biochemistry, and complete blood count analyzer results for the same items were all >0.850.

**Table 1:** Potassium, sodium, glucose, and hemoglobin levels measured by blood gas, dry chemistry, and complete blood count analyzers at the study sites.

**Parameters**	**Hospital**	**Number of** **paired tests**	**Analyzer**	**Range (mix–max)**	**media*n* (P25, P75)**	**correlation coefficients** **(r) [95% CI]**	***P* value**
K (mmol/L)	ZRY hospital	749	ABL800 FLEX	2.0–10.0	3.7 (3.4, 4.1)	0.9573 [0.9506–0.9630]	*P* < 0.0001
			VITROS5600	1.98–10.17	3.81 (3.49, 4.22)		
	QY hospital	2,073	GEM3500	1.1–8.3	3.7 (3.2, 4.1)	0.9198 [0.9126–0.9263]	*P* < 0.0001
			VITROS350	1.2–8.9	4.1 (3.7, 4.6)		
Na (mmol/L)	ZRY hospital	772	ABL800 FLEX	121–175	140 (136, 143)	0.8898 [0.8736–0.9040]	*P* < 0.0001
			VITROS5600	121.5–168.7	138.4 (135.3, 141.9)		
	QY hospital	2,134	GEM3500	95.0–191.0	137.0 (132.8, 142.0)	0.8591 [0.8472–0.8701]	*P* < 0.0001
			VITROS350	100.0–180.0	134.0 (129.0, 137.0)		
Glu (mmol/L)	ZRY hospital	721	ABL800 FLEX	0.9–32.0	8.0 (6.7, 10.20)	0.9849 [0.9825–0.9870]	*P* < 0.0001
			VITROS5600	0.90–32.35	7.72 (6.46, 10.00)		
	QY hospital	486	GEM3500	0.6–29.1	8.2 (6.4, 11.5)	0.9764 [0.9717–0.9803]	*P* < 0.0001
			VITROS350	0.6–27.8	8.5 (6.7, 12.0)		
Hb (g/L)	ZRY hospital	847	ABL800 FLEX	15–202	106 (82, 128)	0.9883 [0.9866–0.9898]	*P* < 0.0001
			BC-6900	16–202	108 (83, 133)		
	QY hospital	2,055	GEM3500	33–198	121 (99, 139)	0.8666 [0.8551–0.8773]	*P* < 0.0001
			XN-9000	33–218	129 (105, 150)		

*K, potassium; Na, sodium; Glu, glucose, Hb, hemoglobin*.

### Bias Analysis of K Measurements

**Figure 1:**
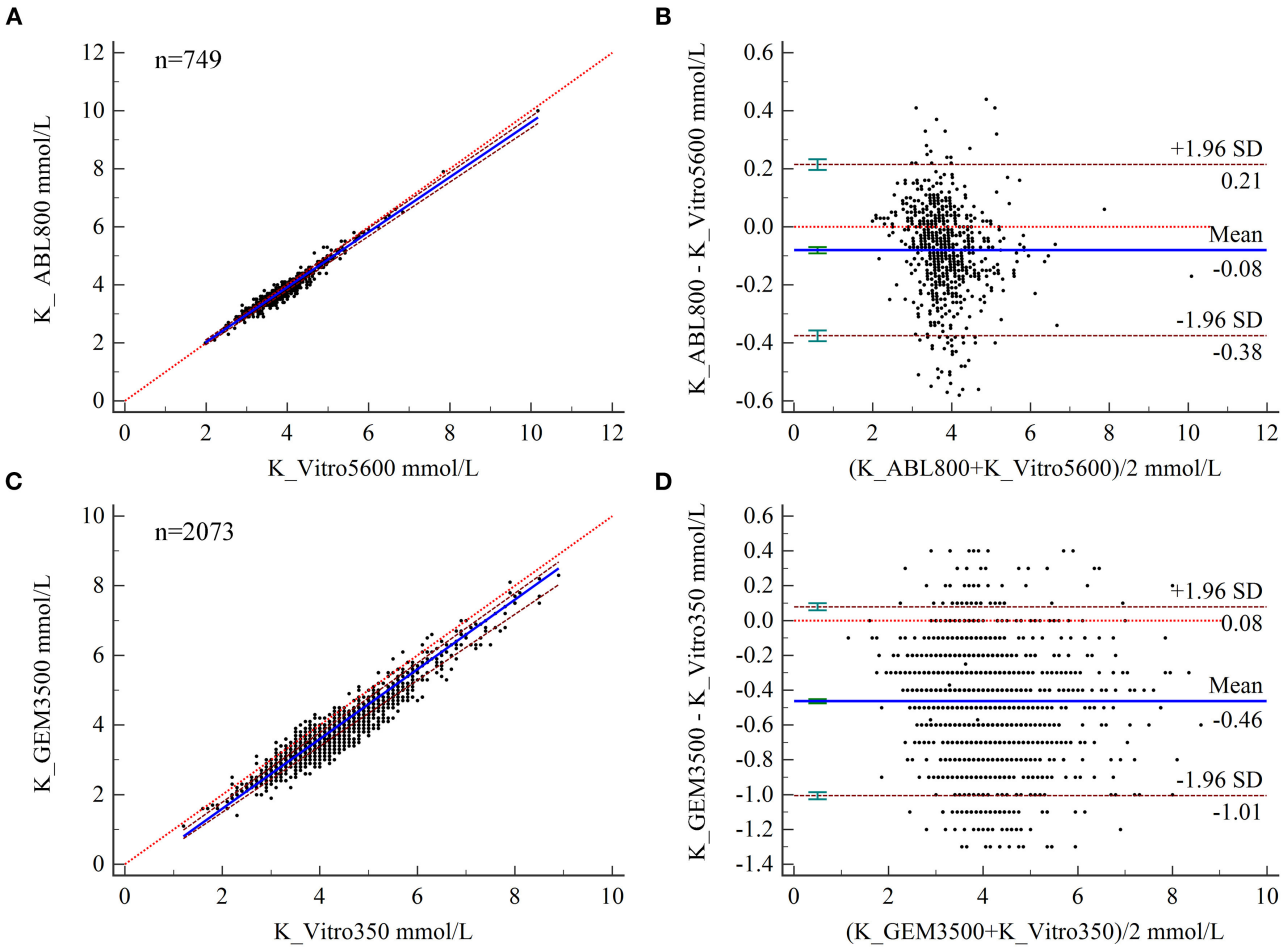
**(A,B)** Scatter diagrams showing the results of Passing–Bablok regression analysis of the comparisons of potassium (K) levels in arterial and venous blood samples between the ABL800 FLEX and VITROS5600 systems **(A)** and between the GEM3500 and VITROS350 systems **(B)**. The graph shows the observations together with the regression line (solid blue line), the confidence interval for the regression line (dashed lines), and the identity line (*x* = *y*, dotted line). **(C, B)** Results of Bland–Altman analysis of the comparisons of K levels in blood and venous serum samples between the ABL800 FLEX and VITROS5600 systems **(C)**, and the GEM3500 and VITROS350 systems **(D)**, showing the 95% limits of agreement (LoA). The scatter diagram shows the differences plotted against the averages of the two measurements. Horizontal lines are drawn at the mean difference and the LoA.

The PB regression model was used to fit the assay results between paired samples (Figure 1A), and the regression equation and 95% CIs for its slope and intercept are shown in Table 2. The expected value (*Yc*) was calculated by substituting the given MDL (*Xc*) into the regression equation separately and compared to ALs to determine the acceptability of the bias. Biochemical analysis was performed at the ZRY Hospital using venous blood plasma samples, with expected biases of K levels measured by BGA of −0.02, −0.17, and −0.26 mmol/L, respectively, for MDLs of 3, 5.8, and 7.5 relative to those measured by biochemical analysis, all of which were below the acceptance criteria. The differences in paired sample measurements were analyzed using the Bland–Altman method (Figure 1B). The mean difference between K levels measured by BGA and biochemistry analyzer at the ZRY Hospital was −0.08 mmol/L (95% CI −0.091 to −0.069), with a 95% LoA of −0.38–0.21 and a mean relative bias of −2.0%. In this analysis, 1.47% (11/749) of paired samples were above the CLIA88 standard, meeting the CLIA88 requirement that ≥80% of samples should show acceptable bias.

**Table 2:** Predicted bias of potassium, sodium, glucose, and hemoglobin at a given medical decision level.

**Parameters**	**Hospital**	**Passing-Bablok regression equation**	**Slope 95% CI**	**Intercept 95% CI**	**MDL (*Xc*)**	**Predicted values (*Yc*)**	**(*Xc+Yc*)/2**	**Absolute bias (mmol/L)**	**Relative bias (%)**	**ALs**	**Clinical acceptability**
K	ZRY hospital	*y* = 0.1347 +	0.9326–0.9615	0.0808–0.1896	3	2.98	2.99	−0.02	−0.77	0.5 mmol/L	Yes
		0.9474x			5.8	5.63	5.71	−0.17	−2.98	0.5 mmol/L	Yes
					7.5	7.24	7.37	−0.26	−3.53	0.5 mmol/L	Yes
	QY hospital	*y* = −0.2996 + 0.9612x	0.9460–0.9764	−0.3626–0.2366	3	2.58	2.79	−0.42	−14.90	0.5 mmol/L	Yes
					5.8	5.28	5.54	−0.52	−9.47	0.5 mmol/L	No
					7.5	6.91	7.20	−0.59	−8.20	0.5 mmol/L	No
Na	ZRY hospital	*y* = −17.3208 + 1.1321x	1.1111–1.1628	−21.6047–14.4444	115	112.87	113.93	−2.13	−1.87	4 mmol/L	Yes
					135	135.51	135.25	0.51	0.38	4 mmol/L	Yes
					150	152.49	151.25	2.49	1.65	4 mmol/L	Yes
	QY hospital	*y* = −6.3846 + 1.0769x	1.0526–1.1000	−9.5000–3.1579	115	117.46	116.23	2.46	2.12	4 mmol/L	Yes
					135	139.00	137.00	4.00	2.92	4 mmol/L	Yes
					150	155.15	152.58	5.15	3.38	4 mmol/L	No
Glu	ZRY hospital	*y* = 0.0269 + 1.0180x	1.0075–1.0286	−0.0577–0.1049	2.5	2.57	2.54	0.07	2.83	10%	Yes
					6.6	6.75	6.67	0.15	2.18	10%	Yes
					10	10.21	10.10	0.21	2.04	10%	Yes
	QY hospital	*y* = −0.0674 + 1.0435x	1.0268–1.0600	−0.2040–0.0750	2.5	2.54	2.52	0.04	1.64	10%	Yes
					6.6	6.82	6.71	0.22	3.27	10%	Yes
					10	10.37	10.18	0.37	3.61	10%	Yes
Hb	ZRY hospital	*y* = −0.4783 + 0.9783x	0.9683–1.0000	−3.0000–0.5397	45	43.54	44.27	−1.46	−3.29	7%	Yes
					105	102.24	103.62	−2.76	−2.66	7%	Yes
					170	165.83	167.91	−4.17	−2.49	7%	Yes
					230	224.52	227.26	−5.48	−2.41	7%	Yes
	QY hospital	*y* = 7.8750 + 0.8750x	0.8571–0.8929	5.6071–10.1429	45	47.25	46.13	2.25	4.88	7%	Yes
					105	99.75	102.38	−5.25	−5.13	7%	Yes
					170	156.63	163.31	−13.38	−8.19	7%	No
					230	209.13	219.56	−20.88	−9.51	7%	No

*K, potassium; Na, sodium; Glu, glucose; Hb, hemoglobin; MDL, medical decision levels; ALs, acceptance limits*.

In contrast, the expected biases of −0.52 and −0.59 mmol/L for K measured by BGA at MDLs of 5.8 and 7.5 at QY Hospital (Table 2) relative to the results of biochemical analysis using venous blood serum were clinically unacceptable. The mean difference in K level measured by the two testing systems was −0.46 mmol/L (95% CI −0.475 to −0.452), with a 95% LoA of −0.08–1.01 and a mean relative bias of −11.9%. In this analysis, 34.42% (672/2,073) of paired samples were above the CLIA88 standard, with a bias coincidence rate <80% (Figures 1C,D).

### Bias Analysis of Na Measurements

**Figure 2:**
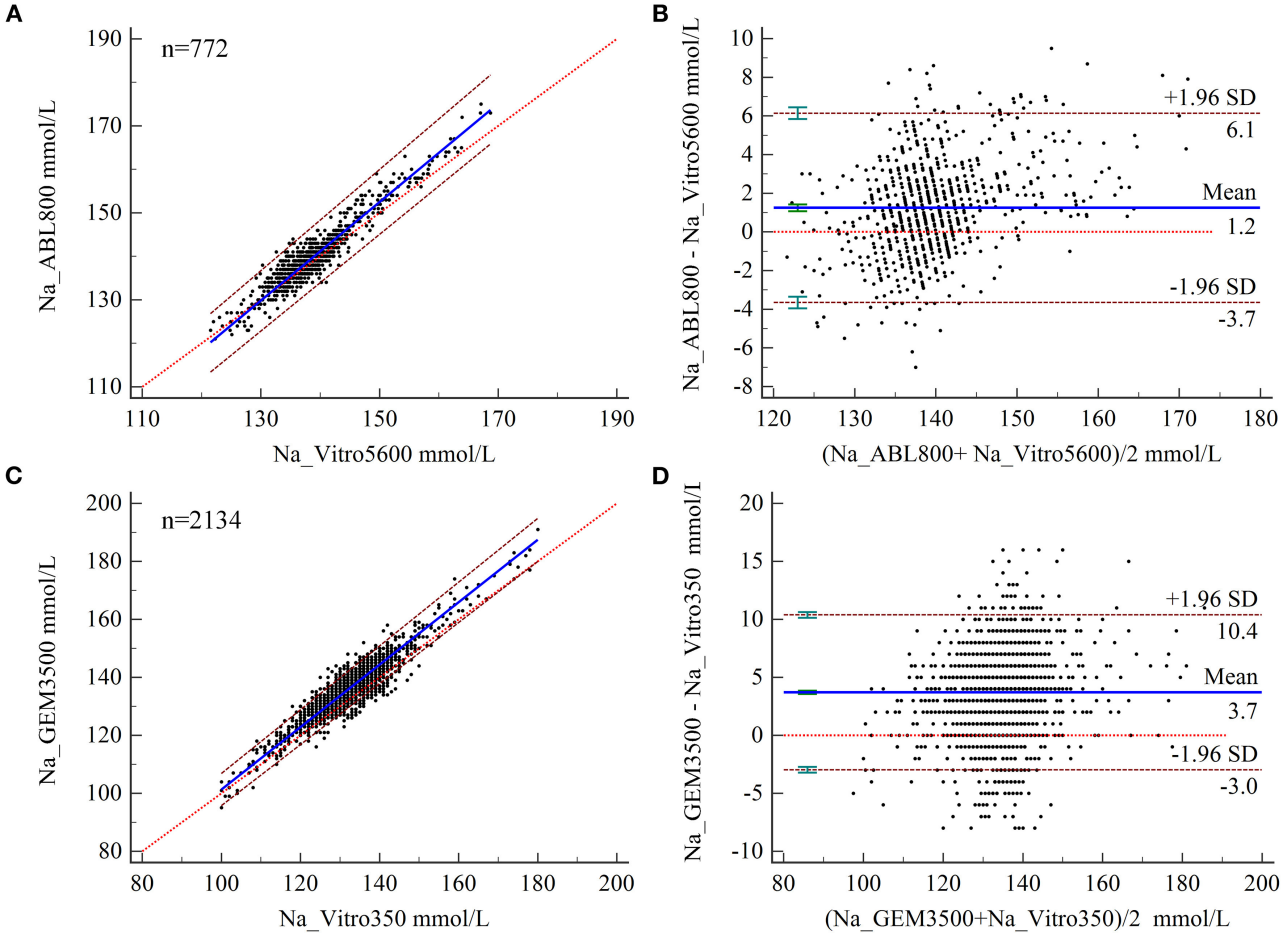
**(A,B)** Scatter diagrams showing the results of Passing–Bablok regression analysis of the comparisons of sodium (Na) levels in arterial blood and venous serum samples between the ABL800 FLEX and VITROS5600 systems **(A)** and the GEM3500 and VITROS350 systems **(B)**. The graph shows the observations together with the regression line (solid blue line), the confidence interval for the regression line (dashed lines), and the identity line (*x* = *y*, dotted line). **(B,C)** Results of Bland–Altman analysis of the comparisons of Na levels measured in blood and venous serum samples between the ABL800 FLEX and VITROS5600 systems **(C)** and between the GEM3500 and VITROS350 systems **(D)** showing the 95% limits of agreement (LoA). The scatter diagram shows the differences plotted against the averages of the two measurements. Horizontal lines are drawn at the mean difference and the LoA.

The expected biases at MDLs of 115, 135, and 150 for Na levels measured by BGA at ZRY Hospital were −2.13, 0.51, and 2.49 mmol/L, respectively, relative to those measured by biochemical analysis (Table 2), all of which were below the acceptance criteria. The mean difference in Na level was 1.2 mmol/L (95% CI 1.06–1.42), with a 95% LoA of −3.7–6.1 and a mean relative bias of 0.9%. In this analysis, 13.99% (108/772) of the paired samples were above the CLIA88 criteria and the bias coincidence rate met CLIA88 requirements (Figures 2A,B).

The expected biases for Na measured by BGA at the QY Hospital were 2.46 and 4.00 at MDLs 115 and 135, respectively, while the expected bias at MDL 150 was 5.15 (unacceptable) (Table 2). The mean difference in Na level measured by BGA compared with that measured by biochemical analyzer was 3.7 mmol/L (95% CI 3.57– 3.85), with a 95% LoA of −3.0–10.4 (**Figure 4B**) and a mean relative bias of 2.7%. In this analysis, 43.44% (927/2,134) of the paired samples were above the CLIA88 criteria, so that the CLIA88 comparison requirements were not met (Figures 2C,D).

### Bias Analysis of Glu Measurements

**Figure 3:**
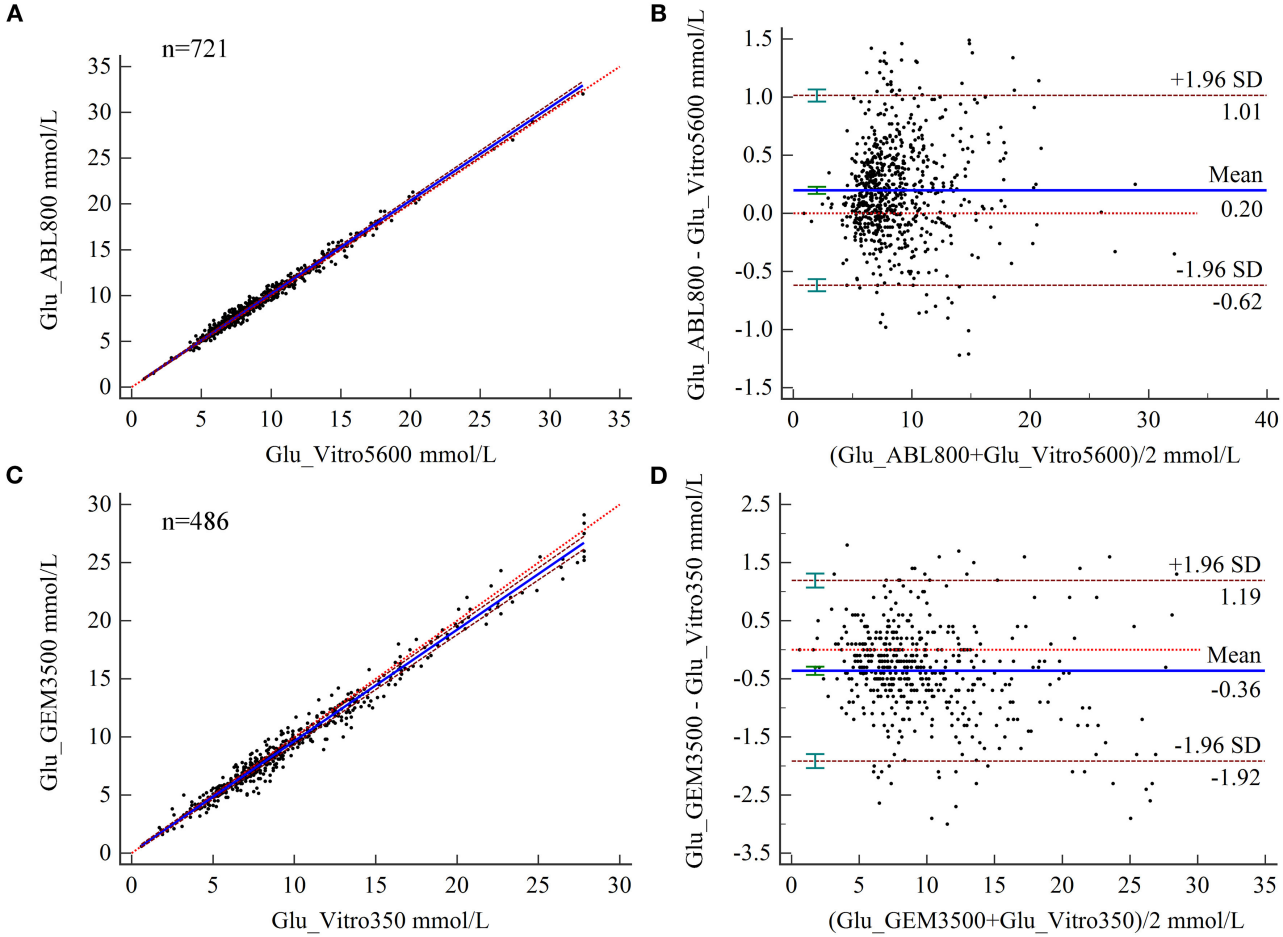
**(A,B)** Scatter diagrams showing the results of Passing–Bablok regression analysis of the comparisons of glucose (Glu) levels in arterial blood and venous serum samples between the ABL800 FLEX and VITROS5600 systems **(A)** and between the GEM3500 and VITROS350 systems **(B)**. The graph shows the observations together with the regression line (solid blue line), the confidence interval for the regression line (dashed lines), and the identity line (*x* = *y*, dotted line). **(C,B)** Results of Bland–Altman analysis comparisons of Glu levels in blood and venous serum samples between the ABL800 FLEX and VITROS5600 systems **(C)** and the GEM3500 and VITROS350 systems **(D)**, showing the 95% limits of agreement (LoA). The scatter diagrams show the differences plotted against the averages of the two measurements. Horizontal lines are drawn at the mean difference and the LoA.

The results of PB regression analysis showed relative biases of Glu measured by BGA at MDLs of 2.5, 6.6, and 10 for the two hospitals of 2.83, 2.18, and 2.04% and 1.64, 3.27, and 3.61%, respectively, all of which were below the acceptable limits (Table 2). Bland–Altman analysis showed a mean difference of 0.20 mmol/L (95% CI 0.167–0.228) for Glu measured by BGA at the ZRY Hospital compared with biochemical analysis, with a 95% LoA of −0.62–1.01 and a mean relative bias of 2.3%. In this analysis, 7.49% (54/721) of the paired samples were above the CLIA88 standard; thus, the CLIA88 matching requirements were met (Figures 3A,B). The mean difference in Glu measurement between the two testing systems at the QY Hospital was −0.36 mmol/L (95% CI −0.433 to −0.291), 95% LoA −1.92–1.19, mean relative bias −3.66%, with 23.05% (112/486) of paired samples above the CLIA88 criterion, which was below but very close to the CLIA88 required coincidence rate (Figures 3C,D).

### Bias Analysis of Hb Measurements

Relative to the Hb results of complete blood count analysis, the relative biases of Hb measured by BGA at the ZRY Hospital at MDLs of 45, 105, 170, and 230 were acceptable (−3.29, −2.66, −2.49, and −2.41, respectively). The Hb level measured by BGA at the QY Hospital at low MDLs of 45 and 105 (4.88 and −5.13, respectively), as well as at high MDLs of 170 of 230 (−8.19 and −9.51, respectively), showed acceptable bias above the AL (Table 2).

**Figure 4:**
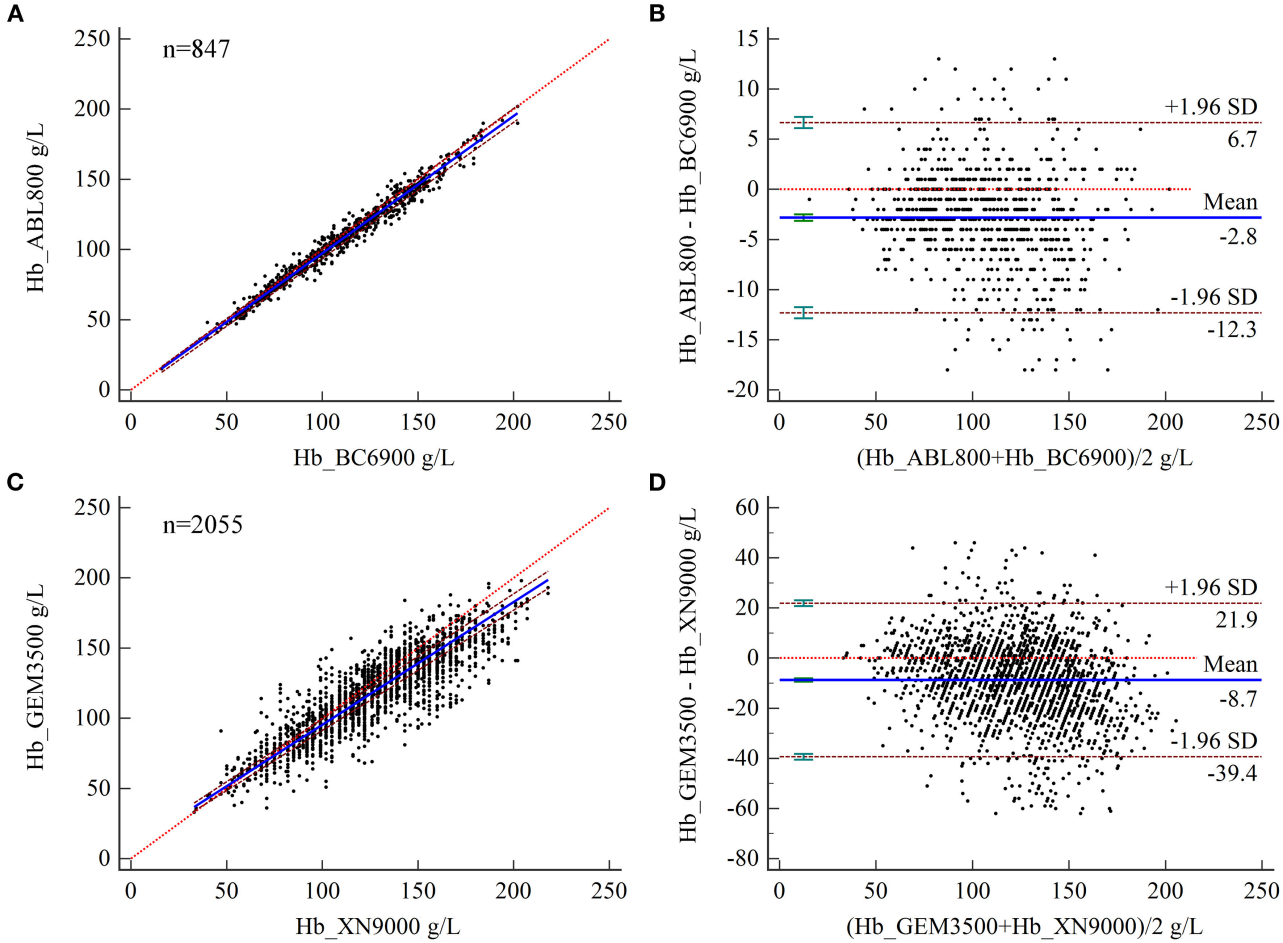
**(A,B)** Scatter diagrams showing the results of Passing–Bablok regression analysis of the comparisons of hemoglobin (Hb) levels in arterial and venous blood samples between the ABL800 FLEX and BC-6900 systems **(A)** and between the GEM3500 and XN-9000 systems **(B)**. The graph shows the observations together with the regression line (solid blue line), the confidence interval for the regression line (dashed lines), and the identity line (*x* = *y*, dotted line). **(C, D)** Results of Bland–Altman analysis of the comparisons of Hb levels in arterial and venous blood samples between the ABL800 FLEX and BC-6900 systems **(C)** and the GEM3500 and XN-9000 systems **(D)**, with 95% limits of agreement (LoA). The scatter diagram shows the differences plotted against the averages of the two measurements. Horizontal lines are drawn at the mean difference and the LoA.

The results of Bland–Altman analysis showed a mean difference in Hb levels measured by BGA and complete blood count analyzer at the ZRY Hospital of −2.8 g/L (95% CI −3.14–−2.49), with 95% LoA of −12.3–6.7 and mean relative bias of −2.7%. In this analysis, 15.70% (133/847) of the paired samples showed high bias confidence according to the CLIA88 criteria (>80%) (Figures 4A,B) The mean difference in Hb between the two testing systems at the QY Hospital was −8.7 g/L (95% CI −9.40- to 8.05), with a 95% LoA of −39.4–21.9, mean relative bias of −6.7%, and 58.15% (1,195/2,055) of the paired samples above the CLIA88 criteria (Figures 4C,D).

## Discussion

The discrepancy between the direct and indirect ISE methods is well established (9, 13, 14). Plasma is ~93% water and 7% solids, and plasma electrolytes are almost entirely related to the aqueous component. The indirect ISE measurement technique uses a predetermined volume of ionic solution to dilute the plasma sample, usually adjusting the assay concentration by a fixed factor of 0.93 to account for the effect of plasma composition. Abnormal concentrations of proteins or other Non-aqueous components can introduce errors by altering the plasma water content (18, 19). An estimated 1/4 of indirect ISE ICU plasma sodium measurements differ from the corresponding direct ISE values by at least 4 mmol/L, the dominant factor being indirect ISE overestimation associated with hypoproteinemia (20). In contrast, the direct ISE method measures plasma electrolyte concentrations without sample dilution, so that changes in plasma water composition do not affect the results. Both BGA and dry biochemistry analyzers use the direct ISE method for measurements. However, the differences between electrolyte concentrations monitored by these two commonly used direct ISE analyzers are much less than those reported between direct and indirect ISE devices. The consistency between the two instruments in the absence of Pre-analytical dilution and independent of total protein concentration is a topic of interest.

We retrospectively analyzed the results of paired samples from two teaching hospitals. At the ZRY Hospital, ABL800 FLEX BGA and VITROS5600 biochemistry analyzer systems were used to measure electrolytes from arterial whole blood and venous blood plasma, respectively. We found that the ABL800 FLEX results for K and Na were interchangeable with those of the VITROS5600, with biases for K and Na at each MDL within the clinically acceptable limits. The results of the Bland–Altman analysis showed a negative bias of 0.08 mmol/L for K measured by the ABL800 FLEX and a very narrow 95% LoA (−0.38–0.21), with only 1.47% of paired samples above the CLIA88 standard. The mean difference for Na was 1.2 mmol/L, with a 95% LoA of −3.7–6.1, with the paired samples meeting the CLIA88 criterion for bias. Although the mean difference in Na was small (1.2 mmol/L, 95% CI 1.06–1.42), the 95% LoA suggested that a small number of samples may have had large differences, which may be due to occasional contamination of some samples with saline rinse solution in the indwelling arterial catheter during sample collection. In another study comparing two direct ISE assay devices, Brigit et al. investigated the concordance between the ABL700 BGA and VITROS (5.1FS and 5,600) biochemistry analyzers for Na detection, reporting a mean difference between arterial and venous plasma Na of 0.087 mmol/L and a 95% LoA of −3.575–3.400 mmol/L (10).

The ZRY Hospital used blood gas syringes and anticoagulant tubes containing dry electrolyte-balanced lithium heparin (DELH). The use of liquid heparin to flush conventional syringes has been reported to cause sample dilution; moreover, heparin itself binds positively charged ions, which can cause Pre-analytical biases in electrolyte concentrations (21). The use of dry electrolyte-balanced heparinized syringes has been shown to reduce negative bias in the measurement of positively charged electrolytes (22).

While the QY Hospital also used this type of syringe, venous blood serum samples were used for the biochemical analyses. The GEM3500 BGA showed significantly lower K concentrations in arterial blood compared to those in venous serum measured by the VITROS350 analyzer. The systematic error between the two systems was clinically acceptable at only 3.0 mmol/L out of three MDLs. A prospective study by Zhang et al. showed that the BGA detected 0.43 mmol/L lower K levels compared to a biochemical analyzer that used the direct ISE principle and venous serum samples, with 88% (44/50) of the paired samples exceeding the CLIA limits (23). The reference range for serum K was 3.8–5.0 mmol/L and that for plasma K 3.5–4.7 mmol/L, with the former concentration being on average 0.3 mmol/L higher than the latter, according to Babic et al. (24). This discrepancy may be mainly related to platelet and leukocyte destruction due to coagulation and centrifugation, as well as Non-dominant hemolysis resulting from red blood cell fragmentation, which leads to higher serum K concentrations.

Similarly, the expected bias in Na levels measured by the GEM3500 BGA at the low MDLs (115 and 135 mmol/L) was clinically acceptable compared to the serum Na concentrations measured by the VITROS350 analyzer. The Bland-Altman analysis showed a mean difference of 3.7 mmol/L and 95% LoA of −3.0–10.4. Zhang et al. reported a mean deviation of 3.04 mmol/L and a 95% LoA of −1.24–7.31 for serum sodium between BGA and ISE-based biochemical analyzer assays (23). We believe that since conditions associated with sodium abnormality are relatively mild, it is sensible to start treatment based on BGA results when they are consistent with the patient's presentation of hyponatremia. However, the broad 95% LoA for both testing systems and the fact that 43.44% of the paired samples were above the CLIA88 standard also affects the safety of the interchangeable use of these measurements in clinical practice.

Whether arterial and venous blood have different levels of electrolytes remains a question worth of discussion. Yan et al. followed a self-paired design to simultaneously detect the levels of routine biochemistry parameters in both venous and arterial plasma using an automated biochemical analyzer (25). The results revealed that statistically significant differences were found for potassium and sodium, but only 14.35 and 2.9% of them were higher than total error, thus meeting the CLIA88 requirement that the proportion of samples with bias within acceptable range should be 80% or higher. Thus, the difference between two blood samples was of no notable clinical significance. Mubina et al. analyzed 110 paired samples between arterial blood gas (ABG) and peripheral venous blood gas (VBG) values, the results indicating that the average discrepancies of sodium and potassium levels between arterial and venous blood were −1.8 mEq/L and −0.04 mEq/L, and limits of agreement (95%) were within acceptable limits. The authors cautiously suggest that venous blood can be used as an acceptable substitute for arterial blood to avoid repeated arterial blood sampling (26). However, when the specimen types are different, we recommend that clinicians continuously monitor electrolytes levels from a single direct ISE source when the tolerance for imprecision is low, such as when correcting hyponatremia.

Accurate Glu values are critical in the management of diabetic emergencies (e.g., hypoglycemia) and maintaining tight glycemic control in critically ill patients, as even mild hypoglycemia can significantly increase mortality (27, 28). Dry biochemistry analyzers use the glucose oxidase method to measure blood Glu while BGA use the amperometric method using an enzyme electrode that contains glucose oxidase to quantify Glu levels. The results of regression analysis showed that the expected deviations at the three MDLs for both assay systems were less than the clinically acceptable range regardless of whether the dry biochemistry analyzer used serum or plasma samples. However, the GEM3500 underestimated blood Glu levels compared to the corresponding biochemistry analyzer, whereas the ABL800 FLEX showed a positive bias. The reasons for these discrepancies are not yet well understood.

Yan et al. reported that there was no statistically significant difference between glucose levels in venous and arterial plasma using an automated biochemical analyzer (25). Further performance validation (proficiency testing) using the 80% principle showed significantly better concordance between the BGA and plasma biochemical test results than between the BGA and serum biochemical test results. The latter had a concordance rate of 76.95%, which is very close to the CLIA88 requirement for interchangeability; thus, it still helps in the clinical assessment of blood Glu increases or decreases. Since arterial blood is an immediate test, the time required for glycolysis by erythrocytes in whole blood is short. In contrast, serum requires at least 30 min for complete clotting before centrifugation. In some specific pathophysiological settings, such as high-dose anticoagulant therapy or the presence of cryoglobulins, the clotting duration may be longer (29). According to reports, glucose concentration decreased markedly beginning from the first hours of storage in plain serum (30). A delay between sample collection and analysis may be an important reason for the discrepancy between the two results. In contrast, plasma samples can have significantly shorter turn-around times, especially during the peak time period of the assay. In addition, Glu differences may also be at least partially related to erythrocyte pressure and pH levels (31, 32). Furthermore, since different amounts of blood were used between BGA and dry biochemistry analyzers, the sample-induced bias deserves further exploration.

Providing early and accurate hemoglobin concentrations is essential for the timely management of patients with hemorrhage, both to provide a baseline assessment of blood loss and to determine transfusion requirements. Thus, this study also investigated the consistency of Hb test results. We found that Hb levels measured by the ABL 800 closely matched those measured by the complete blood count analyzer, with a bias smaller than the clinically acceptable range, a finding consistent with those reported by Zhang et al. and Gibbons et al. (23, 33). These studies compared the ABL-90FLEX and ABL 800 Flex with Sysmex XE-2100 and Sysmex XT1800i complete blood count analyzers, respectively. We found that Hb levels measured by the GEM3500 deviated significantly from those measured by the complete blood count analyzer, which may be related to the detection principle of the instrument. At present, Hb levels are mostly determined by the sodium lauryl sulfate (SLS) colorimetric method, while the reference standard method is the cyanogenic high iron Hb assay. In contrast, the ABL 800 Flex analyzer uses multiwavelength spectrophotometry to determine total Hb. The Hb level measured by the GEM 3500 system was calculated using the formula Hb (g/dL) = HCT (%) ×0.34, in which the hematocrit (HCT) was determined by measuring the electrical conductivity of red blood cells; thus, a deviation in HCT resulted in a systematic error in Hb. In contrast with the reported overestimation of hemoglobin levels reported by Prakash (34) and Allardet-Servent (12), we observed a negative bias in both BGAs performed for comparison, indicating that the BGA measurements overestimated the risk of patients requiring blood transfusion. Therefore, we recommend venous blood to be collected for analysis and confirmation when the results of blood gas analysis are close to the transfusion threshold for critically ill patients (70 g/L).

In summary, the results of our comparative analysis of BGA and dry biochemistry analyzers for the measurement of K, Na, and Glu levels indicated that sample type may be an important factor affecting the consistency of results. In addition, based on our limited data, the consistency between BGA-measured Hb and blood cell assay results may be affected by the principle of BGA testing. Clinicians should be aware of these differences and interpret these parameters with caution in disease management. Our conclusions are limited to our testing equipment and examination procedures, and their generalization requires validation by other laboratories.

## Data Availability Statement

The raw data supporting the conclusions of this article will be made available by the authors, without undue reservation.

## Ethics Statement

This study was approved by the Ethic Committee of Zhejiang Provincial People's Hospital and was conducted according to the declaration of Helsinki.

## Author Contributions

HX: conceptualization, funding acquisition, investigation, methodology, formal analysis, and writing of the original draft. SL and SC: conceptualization, investigation, methodology, formal analysis, and writing of the original draft. DY and JG: methodology and data curation. WJ and WX: investigation, methodology, project administration, writing review, and editing. All the authors have accepted responsibility for the entire content of this manuscript and approved its submission.

## Funding

This work was supported by the National Natural Science Foundation of China (81600110), Natural Science Foundation of Shandong Province (ZR2020MH116), Medical and Health Science and Technology Program of Zhejiang Province (2021KY017 and 2022KY027), and Zhejiang Provincial Department of Education Program (Y202146133).

## Conflict of Interest

The authors declare that the research was conducted in the absence of any commercial or financial relationships that could be construed as a potential conflict of interest.

## Publisher's Note

All claims expressed in this article are solely those of the authors and do not necessarily represent those of their affiliated organizations, or those of the publisher, the editors and the reviewers. Any product that may be evaluated in this article, or claim that may be made by its manufacturer, is not guaranteed or endorsed by the publisher.
