# Supplementary material for: Agreement of Potassium, Sodium, Glucose, and Hemoglobin Measured by Blood Gas Analyzer With Dry Chemistry Analyzer and Complete Blood Count Analyzer: A Two-Center Retrospective Analysis
Source: Front Med (Lausanne). 2022 Apr 1;9:799642. doi: 10.3389/fmed.2022.799642 (PMC9011334; doi:10.3389/fmed.2022.799642)
Supplement: Supplementary file 1 [file Table_1.pdf]

Supplementary Information Table S1 Relevant parameters based on data from equipment manuals

	Devices	*processing time	*blood consumption	operation difficulty
Blood Gas Analyzers	Radiometer ABL800 Flex	24 tests/hour	95μl	Easy, requires operation training
	Gem Premier 3500	85 seconds/per specimen	150μl	Easy, requires operation training
Dry Biochemistry Analyzers	Johnson VITROS*5600*	1300-1500 tests/hour	K:10μl, Na: 10μl, Glu:10μl	Easy, requires operation training
	Johnson VITROS350	300 tests/hour	K:10μl, Na: 10μl, Glu: 10μl	Easy, requires operation training
Complete Blood Count Analyzer	Mindray BC-6900	125 tests/hour	200μl	Easy, requires operation training
	Sysmex XN-9000	100 tests/hour	88μl	Easy, requires operation training

*Relevant data were from the manufacturer's instrument manual
